# The draft genome of the hyperthermophilic archaeon *Pyrodictium delaneyi* strain hulk, an iron and nitrate reducer, reveals the capacity for sulfate reduction

**DOI:** 10.1186/s40793-017-0260-4

**Published:** 2017-08-15

**Authors:** Lucas M. Demey, Caitlin R. Miller, Michael P Manzella, Rachel R. Spurbeck, Sukhinder K. Sandhu, Gemma Reguera, Kazem Kashefi

**Affiliations:** 1 Department of Microbiology and Molecular Genetics, Michigan State University, East Lansing, MI USA; 2 Applied Genomics and Biology Group, Department of CBRNE Defense, Battelle Memorial Institute, Columbus, OH USA; 3 Swift Biosciences INC., Ann Arbor, MI USA; 4 Present address: Natural Resource Ecology Laboratory, Colorado State University, Fort Collins, Colorado, USA

**Keywords:** *Pyrodictium delaneyi* strain Hulk, *Pyrodictiaceae*, Sulfate reducer, Hyperthermophile, Juan de Fuca ridge

## Abstract

*Pyrodictium delaneyi* strain Hulk is a newly sequenced strain isolated from chimney samples collected from the Hulk sulfide mound on the main Endeavour Segment of the Juan de Fuca Ridge (47.9501 latitude, −129.0970 longitude, depth 2200 m) in the Northeast Pacific Ocean. The draft genome of strain Hulk shared 99.77% similarity with the complete genome of the type strain Su06^T^, which shares with strain Hulk the ability to reduce iron and nitrate for respiration. The annotation of the genome of strain Hulk identified genes for the reduction of several sulfur-containing electron acceptors, an unsuspected respiratory capability in this species that was experimentally confirmed for strain Hulk. This makes *P. delaneyi* strain Hulk the first hyperthermophilic archaeon known to gain energy for growth by reduction of iron, nitrate, and sulfur-containing electron acceptors. Here we present the most notable features of the genome of *P. delaneyi* strain Hulk and identify genes encoding proteins critical to its respiratory versatility at high temperatures. The description presented here corresponds to a draft genome sequence containing 2,042,801 bp in 9 contigs, 2019 protein-coding genes, 53 RNA genes, and 1365 hypothetical genes.

## Introduction

The unifying metabolic feature of the first five species described in the family 10.1601/nm.55 *,* in the archaeal order 10.1601/nm.30, was for long their ability to respire sulfur-containing electron acceptors, mainly elemental sulfur (S^0^), thiosulfate (S_2_O_3_^2−^) and sulfite (SO_3_^2−^) [1]. 10.1601/nm.63 1A^T^ reduces nitrate in addition to thiosulfate [2]. Yet the recently described 10.1601/nm.28674 Su06^T^ reduces nitrate and iron but cannot use sulfur or thiosulfate [3].

Here we report on the isolation and genome sequencing and annotation of a novel strain of 10.1601/nm.28674, designated strain Hulk, capable of hyperthermophilic growth with iron, nitrate, and several sulfur-containing compounds. This makes the novel strain Hulk the first hyperthermophilic archaeon known to respire iron, nitrate, and sulfur-containing electron acceptors. Furthermore, strain Hulk is the first member of the 10.1601/nm.55 family able to use formate as an electron donor and carbon source. In addition, it oxidized peptides, an ability only reported for the two obligate peptide organotrophs in the family, 10.1601/nm.58 AV2^T^ [4] and 10.1601/nm.61 PLM1–5^T^ [5]. The ability to oxidize formate and peptides is of special environmental significance, as these are abundant electron donors in hydrothermal marine vent systems [6–9]. Analysis of the draft genome of strain Hulk reveals numerous pathways and genes that allow this archaeon to couple autotrophic and heterotrophic growth with these many electron donors and acceptors.

## Organism information

### Classification and features

**Fig. 1:**
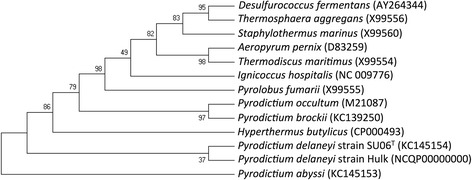
Phylogenetic tree constructed with the maximum likelihood algorithm comparing the 16S rRNA gene sequence from *P. delaneyi*, strain Hulk to the species type strain Su06^T^ and other hyperthermophilic archaea. GenBank accession numbers are listed in parentheses. Bootstrap values displayed at branch points were determined from 100 replicates [72, 73]

A novel strain of 10.1601/nm.28674 designated strain Hulk (Fig. 1) was isolated from a hot sediment sample collected from the Hulk hydrothermal vent located on the Main Endeavour segment of the Juan de Fuca Ridge (47.9501 latitude −129.0970 longitude) in the Northeast Pacific Ocean approximately 300 miles west of Seattle, Washington, at the depth of 2200 m. Enrichment cultures used marine enrichment media [10] modified with the addition NaCl (20 g l^−1^) and yeast extract (25 μg l^−1^), and supplemented with pebble-milled cellulose (0.24%, wt/vol) as the electron donor and poorly crystalline Fe(III) oxide (100 mmol l^−1^) as electron acceptor. The headspace of the tubes was pressurized with N_2_:CO_2_ (80:20%, *v*/v, 101 kPa). Isolation was in the same medium solidified with GELRITE gellan gum (Sigma-Aldrich), as previously described [10]. All incubations during enrichments and isolation were at 100 °C.

Strain Hulk was tested for electron donor/acceptor use in the modified marine medium described above, but incubating the cultures in the dark at the optimum growth temperature (90 °C). The following potential electron donors were tested: H_2_ (H_2_:CO_2_, 80:20%, *v*/v, 101 kPa), lactate (10 mM), pyruvate (10 mM), formate (10 mM), butyrate (2.5 mM), yeast extract (0.01%, wt/vol), methanol (5 mM), ethanol (5 mM), 1-propanol (5 mM), 3-methyl-1-butanol (5 mM), stearate (1 mM), starch (0.1%, wt/vol), D-glucose (5 mM), fructose (5 mM), maltose (2.5 mM), melibiose (2.5 mM), mannose (5 mM), galactose (5 mM), palmitate (1 mM), acetate (10 mM), malate (10 mM), succinate (10 mM), citrate (10 mM), fumarate (10 mM), peptone (0.1%, wt/vol), valeric acid (5 mM), propionate (5 mM), alanine (12 mM), histidine (6 mM), proline (8.7 mM), glycine (14 mM), isoleucine (7.6 mM), aspartic acid (7.6 mM), glutamic acid (6.8 mM), arginine (6 mM), L-cysteine (8 mM), serine (10 mM), asparagine (6 mM), cellulose (0.24%, wt/vol), chitin (0.4%, wt/vol), and cellobiose (0.2%, wt/vol). The electron acceptors tested were: uranium (1 mM), vanadium (1 mM), Fe(III) pyrophosphate (10 mM), sulfate (14 mM), thiosulfate (10 mM), sulfite (4 mM), selenate (10 mM), selenite (10 mM), arsenate (10 mM), nitrate (10 mM), nitrite (1 mM), poorly crystalline iron (Fe[III]) oxides (100 mmol l^−1^), manganese oxides (poorly crystalline Mn[IV], 20 mmol l^−1^), malate (10 mM), fumarate (50 mM), iron (Fe[III]) citrate (50 mM), dimethyl sulfoxide (DMSO, 1 mM), anthraquinone-2,6-disulfonate (AQDS, 5 mM), goethite (50 mmol l^−1^), and hematite (50 mmol l^−1^).

**Fig. 2:**
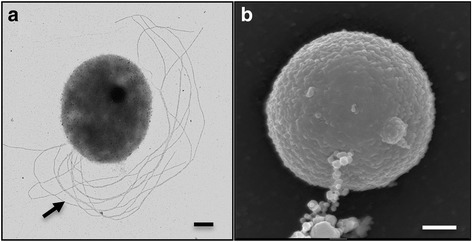
Morphological features. Transmission (**a**) and scanning (**b**) electron micrographs of a cell of strain Hulk grown with formate as the electron donor and Fe(III) citrate and sulfate as the electron acceptors, respectively. Arrow in (**a**) points at the lophotrichous flagella and in (**b**) at membrane vesicles. Scale bars, 200 nm

**Fig. 3:**
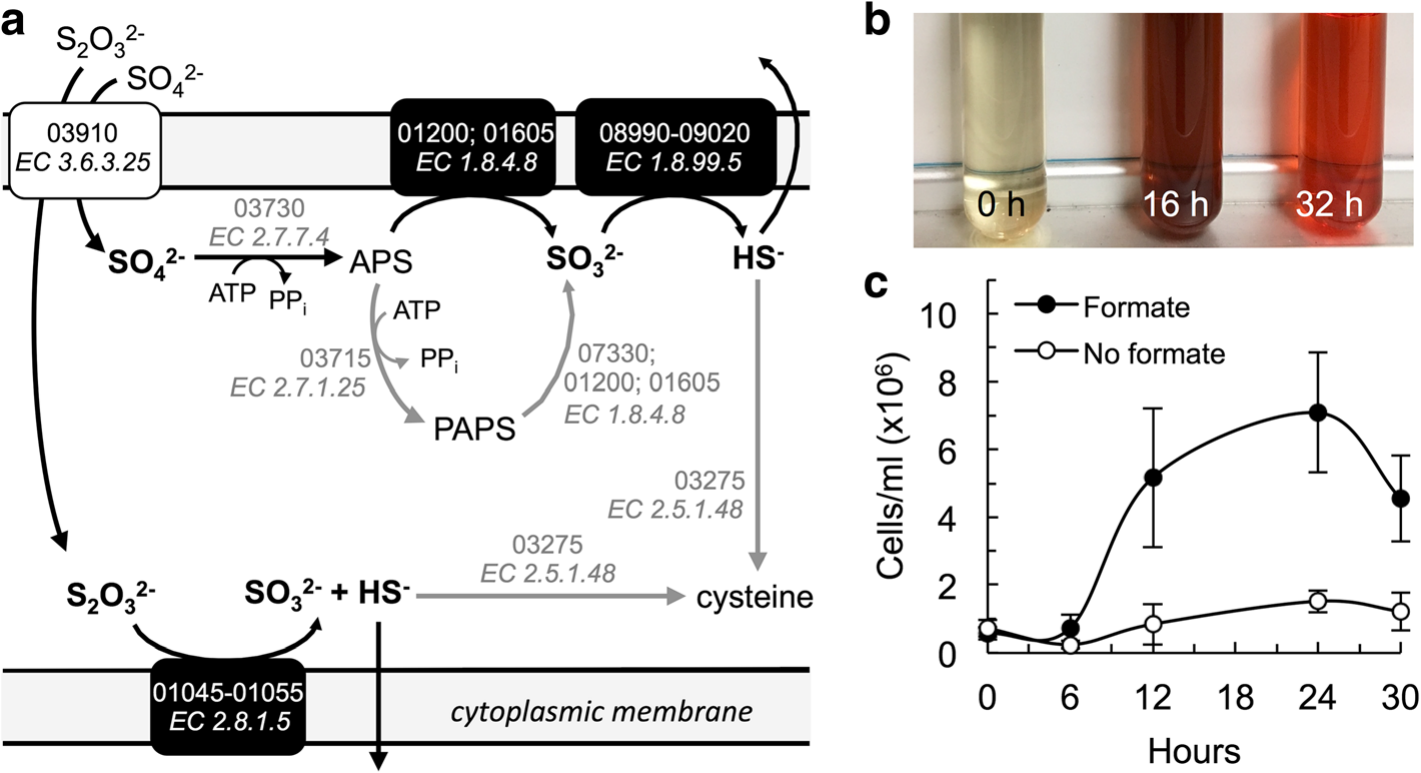
Sulfur metabolism. **a** Predicted pathway for sulfate (SO_4_^2−^) and thiosulfate (S_2_O_3_^2−^) membrane transport and cytoplasmic assimilation (*black arrows*) or dissimilation (*gray arrows*) by strain Hulk. *Dashed arrows* with question marks indicate reaction enzymes lacking obvious matches in the draft genome. The sulfate, thiosulfate, and sulfite (SO_3_^2−^) anions and the sulfide (H_2_S) product are boxed. Annotated ORF and Enzyme Commission (EC) numbers are shown in gray for each reaction enzyme. **b**-**c** Growth with formate and AQDS (**b**) or DMSO (**c**) as electron acceptors. The reduction of AQDS by strain Hulk turns the cultures first *brown*, then *orange* (**b**). The reduction of DMSO supports increases in cell numbers compared to controls without the electron donor (**c**). All cultures were prepared at the optimum pH and salt concentration (7 and 2%, respectively) and incubated at the optimum temperature (90 °C)

Table 1 shows the general features of the novel isolate. The strain was a chemolithoautotroph that coupled the reduction of Fe(III) (provided as poorly crystalline Fe(III) oxides or soluble Fe(III) citrate) to the oxidation of H_2_, formate, peptone, cellobiose, and starch. This contrasts with the type strain Su06^T^, which cannot grow with formate and sugars [3]. Cells of 10.1601/nm.28674 strain Hulk from cultures with formate and Fe(III) citrate or sulfate were cocci of ca. 1.0 μm in diameter, motile, and lophotrichously flagellated (up to 10 flagella per cell were apparent; Fig. 2a). Scanning electron micrographs of cells grown with formate (10 mM) as the electron donor and sulfate (14 mM) as the electron acceptor revealed extensive vesiculation, with some membrane vesicles appearing as filamentous aggregates attached to the cell surface (Fig. 2b). Like strain Su06^T^ [3], strain Hulk also gained energy for growth from the reduction of nitrate. But, unlike the type strain, strain Hulk was capable of growth with most of the sulfur-containing electron acceptors tested such as AQDS, DMSO, sulfate, and thiosulfate (Fig. 3). Also notable is the higher upper temperature limit for growth of strain Hulk (105 °C) compared to the type strain Su06^T^ (97 °C) [3]. Additionally, strain Hulk has a broader range of growth-supporting temperatures (70–105 °C, optimum at 90 °C) and pH (pH 3.3–7.7, optimum at pH 6.7) than strain Su06^T^ (82–97 °C, optimum at 90–92 °C; and pH 0.9–3.6, optimum at pH 1.9) [3]. The range of salt concentrations that supported growth of strain Hulk (0.5–4.5% (*w*/*v*) NaCl, with optima at 2% NaCl) was broader than those reported for the type strain (0.9–3.6% (*w*/*v*), optimum at 1.9% NaCl) [3].

**Table 1:** Classification and general features according to the MIGS recommendations [74]

MIGS ID	Property	Term	Evidence code^a^
	Classification	Domain Archaea	TAS[75]
		Phylum *Crenarchaeota*	TAS[76]
		Class *Thermoprotei*	TAS[77]
		Order *Desulfurococcales*	TAS[78]
		Family *Pyrodictiaceae*	TAS[79]
		Genus *Pyrodictium*	TAS[80]
		Species *Pyrodictium delayeni*	TAS[3]
		Strain: Hulk	IDA
	Gram stain	Not applicable	NAS
	Cell shape	Coccus	IDA
	Motility	Lophotrichous	IDA
	Sporulation	Not applicable	NAS
	Temperature range	70–105 °C	IDA
	Optimum temperature	90 °C	IDA
	pH range; Optimum	3.3–7.7; 6.7	IDA
	Carbon source	CO_2_, formate, peptone, starch and cellobiose	IDA
MIGS-6	Habitat	Marine hydrothermal systems	IDA
MIGS-6.3	Salinity	2.0% NaCl (*w*/*v*)	IDA
MIGS-22	Oxygen requirement	Anaerobic	IDA
MIGS-15	Biotic relationship	Free-living	IDA
MIGS-14	Pathogenicity	Non-pathogen	IDA
MIGS-4	Geographic location	Hulk hydrothermal vent, located on Main Endeavour segment of the Juan de Fuca Ridge, in the Northeast Pacific Ocean approximately 300 miles West of Seattle, Washington, at the depth of 2200 m	IDA
MIGS-5	Sample collection	August 1999	IDA
MIGS-4.1	Latitude	47.9501	IDA
MIGS-4.2	Longitude	−129.0970	IDA
MIGS-4.4	Altitude	Not applicable	NAS

^a^ Evidence codes – IDA: Inferred from Direct Assay; TAS: Traceable Author Statement (i.e., a direct report exists in the literature); NAS: Non-traceable Author Statement (i.e., not directly observed for the living, isolated sample, but based on a generally accepted property for the species, or anecdotal evidence). These evidence codes are from the Gene Ontology project [81]

The draft genome of 10.1601/nm.28674 *s*train Hulk contained a single 16S rRNA gene, whose sequence was 100% identical to the 16S rDNA sequence in the complete genome of 10.1601/nm.28674 strain Su06^T^ [3]. Pair-wise genome comparisons between the two genomes [11] revealed an average nucleotide identity (ANI) of 99.77%, above the cutoff (92%) established for bacterial species definition [12]. The full-length sequence of the 16S rRNA gene of strain Hulk (1496 bp) was used to construct a phylogenetic tree in reference to 16S rRNA gene sequences from other hyperthermophilic archaea (Fig. 1). The 10.1601/nm.61 was the nearest neighbor outside of the 10.1601/nm.28674 species group (99% identity).

## Genome sequencing information

### Genome project history

10.1601/nm.28674 strain Hulk was sequenced and annotated based on its phylogenetic position and its metabolic versatility. Genome comparisons [11] with the recently described species type strain Su06^T^ [3] and other members of the 10.1601/nm.55 were performed to provide novel insights into this archaeal family and the metabolic potential of their members. The draft genome of 10.1601/nm.28674 strain Hulk presented here is contained within 9 contigs with an average coverage of at least 380× (Table 2). This Whole Genome Shotgun project has been deposited at DDBJ/ENA/GenBank under the accession NCQP00000000 (Table 2). The version described in this paper is version NCQP01000000. The genome project summary can be viewed at Genomes Online Database (Ga0169944) [8] (Table 2).

**Table 2:** Genome sequencing project information

MIGS ID	Property	Term
MIGS 31	Finishing quality	9 contigs
MIGS-28	Libraries used	Paired-end
MIGS 29	Sequencing platforms	Illumina
MIGS 31.2	Fold coverage	380×
MIGS 30	Assemblers	Velvet and MIX
MIGS 32	Gene calling method	Gimmer
	Locus Tag	Pdsh_
	Genbank ID	NCQP01000000
	GenBank Date of Release	June 14, 2017
	GOLD ID	Ga0169944
	BIOPROJECT	PRJNA356901
MIGS 13	Source Material Identifier	Hulk (pending assignment by ATCC, DSMZ and JCM)
	Project relevance	Early life, evolution of metal respiration in hyperthermophiles, and marine microbial diversity and ecology

### Growth conditions and genomic DNA preparation

Genomic DNA was extracted from exponentially-grown cells from formate-Fe(III) citrate cultures incubated at 90 °C as previously described [13]. Cells were harvested at room temperature by centrifugation for 20 min, washed with a marine wash buffer [14], and extracted using the MoBio Powersoil kit, except that an additional lysis buffer step was included, as reported elsewhere [14]. Quality of genomic DNA was verified by nanodrop and gel electrophoresis.

### Genome sequencing and assembly

The assembly presented was generated from three paired-end Illumina libraries [15]. One of the libraries was sequenced at the Research Technology Support Facility center at Michigan State University and sequence data for the remaining libraries generated at Swift Biosciences. Genomic DNA was fragmented to an average size of 200 bp using a Covaris M220 (Covaris, Woburn, MA). Two genome libraries were generated using the Accel-NGS 2S DNA Library kit and Accel-NGS 1S DNA Library Kit (Swift Biosciences, Ann Arbor, MI). The third library was generated at the Michigan State University genomics core. These libraries were sequenced on an Illumina MiSeq (Illumina, San Diego, CA) using MiSeq Reagent kit v2. The read data from each of the three libraries were trimmed using fastq-mcf [16] and ConDeTri [17] and analyzed with the genome assembly program Velvet [17] to generate three independent draft de novo genome assemblies. A consensus assembly was then generated using Mix [18] with a minimum alignment length of 200 bp and a minimum contig length of 0 bp. Contigs with total length below 1000 bp were removed from the final assembly. This resulted in an assembly of 9 contigs with an average coverage depth exceeding 380×.

### Genome annotation

The genome annotation used the Rapid Annotations via Subsystems Technology (RAST) server [19]. Coding regions were identified with RAST’s GLIMMER tool [20]. This initial annotation of protein encoding regions in the genome was then manually refined for genes of interest. Selected genes were also analyzed with DELTA-BLAST and PSI-BLAST to identify conserved domains and homology to known proteins and to infer functions. Enzyme Commission numbers and Clusters of Orthologous Group categories were determined with a combination of DELTA-BLAST analysis of each annotated gene and the IMG-ER platform [21]. After COGs were identified the genome was again submitted for annotation via the Prokaryotic Genome Annotation Pipeline and verified the genes referenced here.

Putative *c*-type cytochromes were identified based on the presence of conserved heme-binding motifs (CXXCH), as previously described [13]. The presence of signal peptides or N-terminal helix membrane anchors in the proteins containing the conserved heme-binding motif was then assessed using PRED-TAT [22] and the TMHMM Server (v2.0) [23], respectively, to infer their cellular localization (exported or membrane-bound, respectively). DELTA-BLAST was then used to assess the homology of the putative *c*-type cytochrome proteins with known *c-*type cytochromes in the non-redundant NCBI database.

## Genome properties

The draft genome sequence of 10.1601/nm.28674 strain Hulk was assembled into 9 contigs (*N*50 557,338 bp, total length 2,042,801 bp) with a GC content of 53.88% (Table 3). The draft genome size is close to that reported for the complete genome of the type strain of 10.1601/nm.28674 Su06^T^ (2,023,836 bp) [3]. Out of the total of 2089 genes identified in the genome sequence of strain Hulk, 53 were predicted to encode RNAs and 2019 proteins (Table 3). Seventeen pseudogenes were identified by the Prokaryotic Genomes Automatic Annotation Pipeline [24]. Furthermore, 67.73% of the predicted genes (2089) are represented by COG functional categories. Distribution of these genes and their percentage representation are listed in Table 4.

**Table 3:** Genome statistics

Attribute	Value	% of Total
Genome size (bp)	2,042,801	100
DNA coding (bp)	1,774,506	86.87
DNA G + C (bp)	1,100,639	53.88
DNA scaffolds	9	100
Total genes	2089	100
Protein coding genes	2019	96.65
RNA genes	53	2.54
Pseudo genes	17	0.81
Genes in internal clusters	322	15.41
Genes with function prediction	1415	67.73
Genes assigned to COGs	1234	59.07
Genes with Pfam domains	1428	68.36
Genes with signal peptides	36	1.72
Genes with transmembrane helices	441	21.11
CRISPR repeats	7	0.33

**Table 4:** Number of genes associated with general COG functional categories

Code	Value	%age	Description
J	216	16.22	Translation, ribosomal structure and biogenesis
A	1	0.08	RNA processing and modification
K	58	4.35	Transcription
L	54	4.05	Replication, recombination and repair
B	1	0.08	Chromatin structure and dynamics
D	9	0.68	Cell cycle control, Cell division, chromosome partitioning
V	36	2.7	Defense mechanisms
T	29	2.18	Signal transduction mechanisms
M	26	1.95	Cell wall/membrane biogenesis
N	5	0.38	Cell motility
U	14	1.05	Intracellular trafficking and secretion
O	55	4.13	Posttranslational modification, protein turnover, chaperones
C	109	8.18	Energy production and conversion
G	43	3.23	Carbohydrate transport and metabolism
E	148	11.11	Amino acid transport and metabolism
F	68	5.11	Nucleotide transport and metabolism
H	103	7.73	Coenzyme transport and metabolism
I	36	2.7	Lipid transport and metabolism
P	69	5.18	Inorganic ion transport and metabolism
Q	13	0.98	Secondary metabolites biosynthesis, transport and catabolism
R	176	13.21	General function prediction only
S	58	4.35	Function unknown
-	916	42.6	Not in COGs

The total is based on the total number of protein coding genes in the genome

The preferred start codon in 10.1601/nm.28674 strain Hulk is ATG (45.07%), but the start codons GTG (30.23%) and TTG (24.70%) remain significant. Such start codon preference is similar to that of the type strain Su06^T^ (ATG, 45.08%; GTG, 30.23%, and TTG, 24.69%). The closest relative, 10.1601/nm.61, also had ATG as the preferred codon (38%), followed closely by TTG (37%), then GTG (25%). Another close relative, 10.1601/nm.39 *,* had a preference for the TTG codon (52%), followed by ATG (28%) and GTG (20%) [25, 26]. The draft genome of strain Hulk also contained one SSU RNA (Pdsh_09290), one LSU RNA (Pdsh_09285) and two copies of the 5S rRNA gene (Pdsh_05305 and Pdsh_07510). Table 3 shows additional genome statistics.

Archaeal origins of replication are often AT rich, contain one or more DNA unwinding elements, are in intergenic regions, and possess binding sites for origin binding proteins such as Cdc8 and Orc1 [27]. We identified in the genome of 10.1601/nm.28674 strain Hulk two regions (contig 2, 205,575–205,655; and contig 1, 179,501–179,576) homologous to OR sequences in the DoriC database [28] assigned to 10.1601/nm.10785 (10.1601/strainfinder?urlappend=%3Fid%3DORI+10010050) and 10.1601/nm.66 *islandicus* (10.1601/strainfinder?urlappend=%3Fid%3DORI+10010117). The draft genome of strain Hulk also contained homologs of the DNA replication initiator proteins Cdc6 (Pdsh_04070) and Orc1 (Pdsh_05200), though none were located downstream of either of the predicted origins of replication, as is typical in other archaeal origins of replication [27]. Genes related to cell division and chromosome replication were identified in nearby locations (Pdsh_04035, Pdsh_05165, Pdsh_05230, and Pdsh_05215–05220).

## Insights from the genome sequence

### Central metabolism

**Fig. 4:**
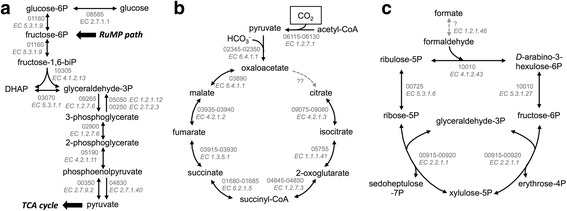
Central metabolism. EMP pathway (**a**), TCA cycle (**b**), and ribulose monophosphate (RuMP) pathway (**c**) predicted from the draft genome of strain Hulk. Abbreviations: P, phosphate; DHAP, dihydroxyacetone phosphate. The annotated ORF number (*top*) and corresponding EC number (*bottom, italicized*) are shown in *gray*. *Dashed gray* arrows indicate reactions catalyzed by enzymes not annotated in the draft genome

Consistent with the ability of strain Hulk to use with glucose-containing sugars such as cellobiose, (a β(1,4) glucose disaccharide) and starch (an α-glucan) as electron donors, we identified in the annotated draft genome genes encoding proteins for the oxidation and assimilation of glucose through a modified Embden-Meyerhof-Parnas glycolytic pathway (Fig. 4a). As is typical in many archaea [29], the modified EMP pathway replaces the glycolytic glyceraldehyde-3-phosphate dehydrogenase and phosphoglycerate kinase enzymes with a glyceraldehyde-3-phosphate ferredoxin oxidoreductase (Pdsh_09265) for the conversion of glyceraldehyde-3-phosphate into 3-phosphoglyceric acid [29]. Interestingly, the pathway is also present in the type strain *P. delayeni* Su06^T^, which, unlike strain Hulk, does not reportedly use sugars as electron donors and carbon sources [3]. The genome of strain Hulk also contains homologs of glyceraldehyde-3-phosphate dehydrogenase (Pdsh_05050) and phosphoglycerate kinase (Pdsh_04830) enzymes, which could function during gluconeogenesis.

Genome analyses also identified most of the enzymes of the tricarboxylic acid cycle (Fig. 4b). The genome encoded a pyruvate carboxylase (Pdsh_02345–02350), which fixes carbon as the bicarbonate anion (HCO_3_^−^) to convert pyruvate into oxaloacetate. However, it lacked the citrate synthase enzyme that catalyzes the condensation of oxaloacetate and acetyl-CoA to generate citrate [30]. It is unlikely that the absence of a citrate synthase was the result of the incomplete genome coverage because genomes from other members of the family 10.1601/nm.55 also lacked obvious citrate transporters (Table 5). Furthermore, the gene is also absent in the complete genomes of most other members of the order 10.1601/nm.30 (Table 5).

**Table 5:** Presence (+) or absence (−) of citrate transporter and synthase proteins

Genome^a^	1	2	3	4	5	6	7	8	9	10
Citrate transporter	–	–	–	+ ^*b*^	+ ^*c*^	+ ^*d*^	+ ^*e*^	–	–	+ ^*g*^
Citrate synthase	–	–	–	–	–	–	–	+ ^*f*^	–	–

^*a*^Sequenced genomes of members of the family *Pyrodictiaceae* (1–3) and other members of the order *Desulfurococcales* (4–10). 1, *P. delaneyi* strain Hulk; 2, *P. delayeni* strain Su06^T^; 3, *Hyperthermus butylicus* PLM1–5^T^; 4, *Pyrolobus fumarii* 1A^T^; 5, *Ignococcus hospitalis* KIN4/I^T^; 6, *Staphylothermus hellenicus* DSM12710^T^; 7, *Desulfurococcus kamchatkensis* 1221n^T^; 8, *Aeropyrum pernix* K1^T^; 9, *Thermosphaera aggregans* M11TL^T^; 10, *Thermogladius cellulolyticus* 1633

^b-g^Protein ID: ^*b*^ WP_014026608.1; ^*c*^ WP_012122975.1; ^*d*^ WP_013142474.1; ^*e*^WP_048058740.1; ^*f*^ BAA80714.1; ^g^ WP_014737180.1

**Fig. 5:**
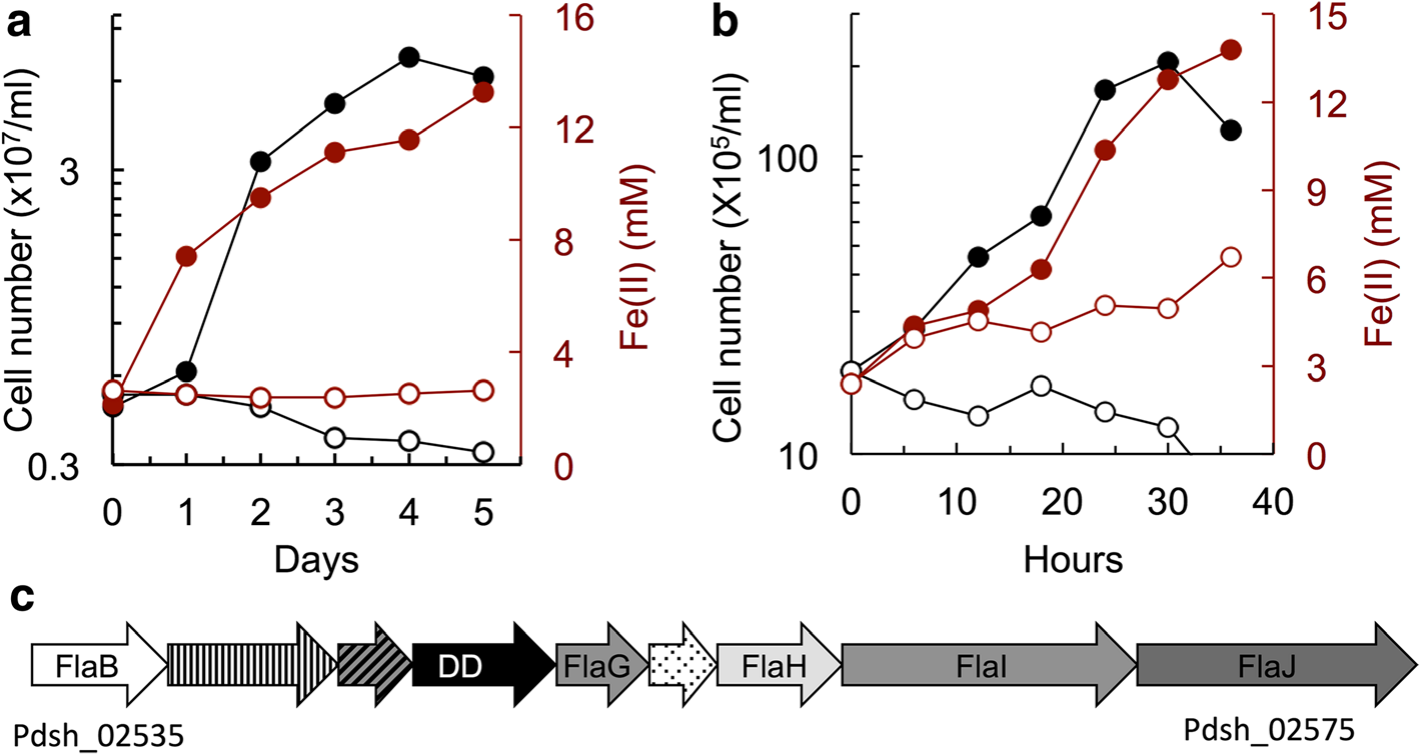
Iron metabolism and motility. **a**-**b** Growth (cell numbers, *black*) and Fe(II) accumulation (*maroon*) of P. delaneyi strain Hulk in cultures with H_2_ and poorly crystalline Fe(III) oxides (**a**) or with formate and Fe(III) citrate (**b**). No donor controls (*open symbols*) are also shown. Each data point shows the average of two biological replicates. **c** Archaellum gene cluster in the draft genome of strain Hulk. (DD, Death Domain superfamily protein)

Interestingly, we identified in the genome of strain Hulk transporters for acetate (ActP acetate permease, Pdsh_04815), a carbon source that, though common in hydrothermal environments [9, 31, 32], does not serve as electron donor for strain Hulk. Furthermore, the genome contained an enzyme for the conversion of acetate into acetyl-CoA (acetyl-CoA synthetase, Pdsh_04765) and several proteins involved in coenzyme A biosynthesis (Pdsh_01535, Pdsh_01545–01560, Pdsh_03865, Pdsh_03830–03880, Pdsh_08205, and Pdsh_08280). We also identified a pyruvate carboxylase enzyme (Pdsh_06115–06130), which fixes CO_2_ in a reaction that converts acetyl-CoA into pyruvate to feed into the TCA cycle (Fig. 4b). This suggests that acetate carbon is assimilated in the TCA cycle yet it is not oxidized because it lacks a citrate synthase. Instead, the TCA cycle can run in reverse, that is, through reductive reactions from oxaloacetate to malate and so on (Fig. 4b). This reverse mode bypasses the need for the citrate synthase step and also enables cells to fix CO_2_. This could explain why strain Hulk grows autotrophically with H_2_ and CO_2_ (Fig. 5a) although its genome lacks key enzymes in other carbon fixation pathways such as the dicarboxylate/4-hydroxybutyrate cycle (DC/HB), the 3-hydroxypropionate/4-hydroxybutyrate cycle (HP/HB), the fuchs-holo bi-cycle, the Calvin–Benson–Bassham cycle, and the Wood-Ljungdahl pathway. Indeed, the reverse TCA cycle has been proposed to be the major autotrophic pathway in hydrothermal vent environments [33].

### Ribulose monophosphate pathway

The pentose phosphate pathway allows many bacteria and eukaryotes to generate reducing power and precursor metabolites needed for the synthesis of nucleotides and aromatic amino acids [29, 34]. However, archaea are not known to have a complete oxidative PPP, and only 10.1601/nm.143 and 10.1601/nm.359 species have a complete non-oxidative PPP [29]. We identified an incomplete non-oxidative PPP in the genome of strain Hulk, but were unable to find homologs of any of the enzymes involved in the oxidative PPP. As is common within the Archaea, a combination of the incomplete non-oxidative PPP and ribulose monophosphate pathway replaces the oxidative PPP to provide reactions for the synthesis of nucleotide and aromatic amino acid precursors such as ribose-5-phosphate and erythrose-4-phosphate respectively [34]. Indeed, the genome of strain Hulk contains all of the genes of the ribulose monophosphate pathway, including a bifunctional D-arabino-3-hexulose 6-phosphate formaldehyde lyase/6-phospho-3-hexuloisomerase (HPF/PHI) and ribose 5-phosphate isomerase (Pdsh_00725) (Fig. 4c).

### Hydrogen and formate as an electron donor during autotrophic growth

**Fig. 6:**
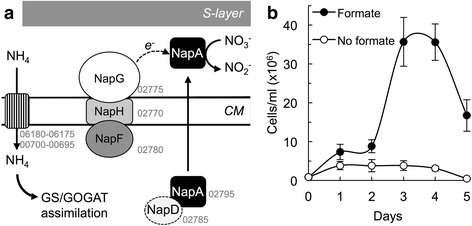
Nitrogen metabolism. **a** Genomic predictions for ammonium (NH_4_) assimilation and Nap pathway for the reduction of nitrate (NO_3_^−^) to nitrite (NO_2_^−^) in strain Hulk. (CM, cytoplasmic membrane). **b** Growth (cell numbers per ml) of strain Hulk with formate and nitrate at optimum conditions of temperature (90 °C), pH (7), and osmolarity (2% NaCl) in reference to controls without the electron donor (formate)

H_2_ and formate are abundant electron donors in hydrothermal vent environments, due to their continuous replenishment through intense serpentinization [6, 9, 35]. These environments are also rich in Fe(III) minerals, creating conditions optimal for the growth of hyperthermophilic iron reducers with H_2_ and formate [10, 13, 36–38]. Indeed, we demonstrated autotrophic growth with H_2_ for strain Hulk (Fig. 5a) and growth with formate during the reduction of sulfur-containing electron acceptors (Fig. 3), Fe(III) citrate (Fig. 5b) and nitrate (Fig. 6) [39].

Consistent with the experimental results, we identified in the genome of strain Hulk a formate dehydrogenase gene cluster (Pdsh_ 05790–05805) encoding two formate dehydrogenase formation proteins (FdhE and FdhD), a putative formate transporter (FdhC), and the formate dehydrogenase α (FdhA) and β (FdhB) subunits. We also identified in the genome of strain Hulk genes encoding the small (Pdsh_08510) and large (Pdsh_09845) subunits of a NiFe hydrogenase, which catalyzes the reversible oxidation of H_2_ [40]. Interestingly, the small NiFe hydrogenase subunit of strain Hulk (Pdsh_08510) did not contain the FeS domains that are needed to transfer electrons from the enzyme center to the electron-accepting substrate [40, 41]. Yet an FeS-containing protein encoded a downstream gene (Pdsh_08500) could mediate this reaction.

The genome of strain Hulk also contained several of the proteins (HypABCDEF) required for the maturation of NiFe hydrogenases [42, 43]. We identified, for example, the hydrogenase maturation protein HypA (Pdsh_01645 and Pdsh_04600) as well as a cluster containing the HypCDEF proteins (Pdsh_08530–08545). Interestingly, neither strain Hulk nor the type strain Su06^T^ contained homologs of HypB, a maturation protein that scavenges nickel at low concentrations for its incorporation in the active site of the NiFe hydrogenase [44]. The loss of HypB can be overcome if high levels of nickel are provided in the external environment [45]. This suggests that the nickel concentrations may be high in hydrothermal environments. Alternatively, yet to be identified nickel-scavenging proteins may be used for the maturation of NiFe hydrogenases in these archaea.

The oxidation of H_2_ or formate by membrane-bound dehydrogenase enzymes contributes to the formation of a proton gradient, which is harnessed to generate energy for growth. Two NADH ubiquinone oxidoreductase (Nuo) proteins could boost the proton motive force. These proteins are encoded in two gene clusters, the first encoding subunits A-D and H-N (Pdsh_06005–05955) and the second coding subunits B-D, L-N and I (Pdsh_06660–06630). NADH ubiquinone oxidoreductase, also known as “respiratory complex 1” [40], is a proton pump able to transfer electrons from NADH to ubiquinone. As a result, the enzyme generates a proton gradient that can be harnessed to synthesize ATP [40].

### Cellobiose and starch as electron donors and carbon sources

In contrast to the type strain Su06^T^, which cannot use sugars as sole electron donors or carbon sources for growth [3], strain Hulk grew with cellobiose and starch as sole electron donors. Consistent with this, the genome of strain Hulk codes for a cellobiose phosphorylase (Pdsh_03720). This enzyme catalyzes the phosphorolysis of the cellulose disaccharide, thus conserving the energy in the β(1,4) glycosidic bond to generate glucose 1-phosphate [46]. Searches for homologs of the Pdsh_03720 cellobiose phosphorylase in the sequenced genomes of other members of the 10.1601/nm.30 only identified a protein with a low degree of homology (66% identity, 84% similarity) in 10.1601/nm.40 (BAN90220.1). By contrast, starch-degrading enzymes were widespread within the 10.1601/nm.30 order. The genome of strain Hulk contained, for example, homologs of a glycosidase (Pdsh_09725) and an amylopullulanase (Pdsh_09650) enzyme. Glycosidase enzymes break the α(1,4) glycosidic bonds in the linear glucose strands of the polymer, whereas amylopullulanases also cleave the branched α(1,6) glycosidic linkages [47]. The genome of strain Hulk also encoded for several glycosyltransferases (Pdsh_02755 and Pdsh_03780), which are enzymes that catalyze the transfer of sugar moieties from activated donor molecules to specific acceptor molecules [48]. It is interesting to note that neither cellobiose nor starch are abundant in most deep-sea environments [49]. Yet, the ability to scavenge these complex sugars has been proposed to allow cells to store carbon as glycogen [49]. However, the genome of strain Hulk and other members of the order 10.1601/nm.30 lack obvious glycogen synthase genes. This could indicate that these organisms carry highly divergent glycogen synthases or that carbon is stored as polymers other than glycogen. Alternatively, yet to be identified sources of complex carbon compounds may be available in these hydrothermal vent environments that select for genomes containing genes involved in the oxidation and assimilation of complex sugars.

### Metabolism of peptones

The availability of free peptides in hydrothermal marine vent systems [7, 8] provides an abundant source of carbon and energy for microorganisms inhabiting these ecosystems. Indeed, peptone served as electron donor to support the growth of strain Hulk. However, growth was not supported with individual amino acids such as histidine, cysteine, and leucine as electron donors. The genome of strain Hulk contains three gene clusters that encode ABC branched amino acid transporters (Pdsh_10390–10,365, Pdsh_01510–01495, and Pdsh_07600–07575). Two gene clusters were identified that encode ABC oligopeptide transporters (Pdsh_05670–05665 and Pdsh_07635–07620), and each oligopeptide transport cluster had dipeptide transport subunits directly upstream (Pdsh_05675 and Pdsh_07640–07645). We also identified in the genome 13 putative peptidases, including a metallocarboxypeptidase (M32) (Pdsh_06240), three aminopeptidases (Pdsh_04150, Pdsh_05315, and Pdsh_07085), a metalloprotease (M50) (Pdsh_05275), and several omega peptidases (Pdsh_06520, Pdsh_06735, Pdsh_07355, Pdsh_07465, and Pdsh_07745). We also identified prolyl oligopeptidases (Pdsh_07465 and Pdsh_07695), an isoaspartyl aminopeptidase (Pdsh_07355), pyrrolidone-carboxylate peptidases (Pdsh_06520 and Pdsh_06735), an exoaminopeptidase (M42) (Pdsh_02360), and a D-aminopeptidase (M55) (Pdsh_08590). The arsenal of peptidase enzymes encoded in the genome of strain Hulk likely maximizes the efficient utilization of the peptides available at hydrothermal sites. Analysis of the complete genome of the type strain Su06^T^ also identified all of these genes, suggesting that the ability to grow with peptones is not restricted to strain Hulk only.

### Iron respiration

The ability of strain Hulk to respire both insoluble (i.e. ferrihydrite) and chelated (i.e. Fe(III) citrate) forms of iron (Fig. 5) further confirms this is a widespread metabolic ability of hyperthermophilic organisms [1]. Mechanistic studies in the model hyperthermophilic archaeon 10.1601/nm.413 demonstrated the need for direct contact between the cell and the iron mineral to transfer respiratory electrons, a reaction that is mediated by *c*-type cytochromes on the outer surface of the cell [13]. Supporting a similar mechanism, we identified in the genome of strain Hulk five genes carrying the conserved heme-binding motif (CXXCH) of *c*-type cytochromes (Pdsh_01720, Pdsh_01730, Pdsh_08690, Pdsh_09040, and Pdsh_10070) (Table 6). All of these genes encoded proteins with signal peptides, as expected of proteins that are exported across the membrane, and none had homology to any known *c*-type cytochromes in the non-redundant NCBI database. The genome also contains 9 genes encoding homologues of the Ccm cytochrome maturation pathway (CcmABCDEFGHI). We identified, for example, *ccmA* (Pdsh_08980), *ccmB* (Pdsh_09025), *ccmC* (Pdsh_09010), and *ccmF* (Pdsh_01745 and Pdsh_09015). Although this cytochrome-*c* maturation system is found in many archaea [50, 51], two of its components (CcmE and CcmH) are often missing or are highly divergent in archaeal genomes [52]. Indeed, the annotation of the genome strain Hulk did not identify a CcmE homolog but included one gene (Pdsh_09020) encoding a protein of the CcdA superfamily that is predicted to be a functional homolog of CcmH [50].

**Table 6:** Strain Hulk’s heme-binding proteins with homologs (>90% identity) in strain Su06^T^

Heme-binding proteins	1	2	3	4	5
Heme motifs	12	8	2	8	2
Molecular weight (kDa, with hemes)	52.5	76.8	77.4	98.1	35.5

Strain Hulk ORF (Su06^T^ protein ID): 1, Pdsh_01720 (ALL00836.1); 2, Pdsh_01730 (WP_055408420.1); 3, Pdsh_08690 (WP_055407817.1); 4, Pdsh_09040 (WP_055410651.1); 5, Pdsh_10070 (WP_055409187.1)

To maximize access to the iron minerals, microorganisms with a direct contact mechanism of electron transfer often rely on flagellar motility and chemotaxis to access the minerals [53]. Although most members of the family 10.1601/nm.55 are reportedly not motile [54], cells of strain Hulk were motile and assembled several flagella (Fig. 2a). A cluster of genes (Pdsh_02535–02575) was annotated in the genome that comprised the minimal gene set (*flaB*, *flaH*, *flaI*, and *flaJ*) needed to assemble the archaeal flagellum or archaellum [27, 55–57] in addition to genes encoding for hypothetical proteins [(Pdsh_02550–02570) (Fig. 5c)]. The protein encoded by Pdsh_02555 had conserved domains described in the archaeal-flagellum superfamily and also had weak homology (27% identify, 44% similarity) to FlaG in 10.1601/nm.20073 (AFH42914.1), a protein proposed to anchor the flagellar hook and filament [58]. The protein encoded by Pdsh_02560 belongs to the DD superfamily that has been implicated in the selective signaling of several complexes [59]. The presence of *flaB*, *flaG*, *flaH*, *flaI*, and *flaJ* homologs and the absence of strong hits to *flaC*, *flaD* and *flaF* is consistent with the type Fla2 gene cluster that is typical of most 10.1601/nm.2 archaella [60]. However, the genome of strain Hulk did not contain any obvious chemotaxis genes. Furthermore, a search in the sequenced genomes within the order 10.1601/nm.30 only identified chemotaxis genes in two species (10.1601/nm.46 and 10.1601/nm.10019). The lack of chemotaxis genes has been proposed to reflect an adaptive response of organisms to hydrothermal environments where intense fluid circulation replenishes electron acceptors and donors [13] Indeed, fluid circulation at the Hulk sulfide chimney vent is high [61]. Thus, the lack of chemotaxis genes within the genome of strain Hulk is consistent with lack of evolutionary pressure to sense chemical gradients.

### Assimilatory and dissimilatory nitrogen metabolism

Nitrogen-containing compounds such as ammonium, nitrate, and nitrite are key intermediates in the marine nitrogen cycle and available for assimilation and respiration in anoxic marine environments [62–64]. Archaea, including members of the 10.1601/nm.2, also contribute to the cycling of nitrogen in deep submarine ecosystems [65, 66]. Consistent with the availability of ammonium in these environments, the genome of strain Hulk contained two ammonium transporters clustered next to a nitrogen regulatory P-II protein (Pdsh_06180–06175 and Pdsh_00700–00695). Also present are a glutamine synthase (Pdsh_02890) for the cytoplasmic incorporation of ammonium into glutamine using α-ketoglutarate as a substrate and a glutamate dehydrogenase (Pdsh_05430), which uses NADPH with α-ketoglutarate and ammonium to produce glutamate [67]. The two enzymes form the GS/GOGAT pathway for the cytoplasmic assimilation of nitrogen from ammonium [67]. The genome also contained an NADPH-dependent glutamate synthase (Pdsh_04375), which converts glutamine into glutamate [67].

By contrast, the genome of strain Hulk lacked nitrate or nitrite transporters for the assimilation of these oxidized nitrogen species. We identified instead homologs of the genes encoding the periplasmic bacterial, dissimilatory nitrate reductase Nap complex (NapADFGH) (Pdsh_02795 and Pdsh_02785–02770) (Fig. 6a). Consistent with the genome prediction, 10.1601/nm.28674 strain Hulk also grew with nitrate as an electron acceptor (Fig. 6b). Also absent in the genome was an obvious homolog of a nitrite reductase. The closest gene (Pdsh_01730) encodes a putative cytochrome-*c*_*7*_ protein containing an NrfH domain. In agreement with the genome prediction, strain Hulk did not use nitrite as an electron acceptor. This suggests that the strain reduces nitrate to nitrite but cannot carry out the complete denitrification of nitrate to N_2_ reported for the type strain Su06^T^ [3].

### Respiration of sulfur-containing electron acceptors

The annotation of the genome of strain Hulk revealed genes encoding proteins involved in assimilatory and dissimilatory metabolism of several sulfur-containing compounds (Fig. 3c). A manual search identified, for example, two genes (Pdsh_01045 and 01050) encoding homologs of the DmsA (25% identity, 41% similarity) and DmsB (29% identity, 42% similarity) subunits of the DMSO reductase complex of the iron-reducing bacterium 10.1601/nm.2931 (SO1429 and SO1430, respectively) [68]. However, the genome of strain Hulk did not contain a homolog of the membrane-bound DmsC protein that anchors the DMSO reductase complex to the periplasmic side of the inner membrane in this bacterium, consistent with the different biochemical composition of the bacterial and archaeal membranes. Yet strain Hulk was able to reduce AQDS and DMSO (Fig. 3a-b).

Also annotated was a multifunctional sulfate/thiosulfate ATP-binding transport protein (Pdsh_03910) for the transport of sulfate (SO_4_^2−^) and thiosulfate (S_2_O_3_^2−^) anions inside the cell (Fig. 3c). The genome also contained a sulfate adenylyltransferase (Pdsh_03730) for the ATP-adenylation of sulfate into adenosine-5′-phosphosulfate (APS), a key step in both the assimilatory and dissimilatory sulfate reduction pathways [69, 70]. Once sulfate is activated, the assimilatory pathway converts APS to 3′-phosphoadenosine 5′-phosphosulfate (PAPS) via an adenylyl sulfate kinase (Pdsh_03715) [69]. PAPS is subsequently converted to sulfite (SO_3_^2−^) by a phosphoadenosine phosphosulfate reductase (PAPS-R) (Pdsh_07330) [69, 71]. Sulfite can also be produced in energy-generating reactions of the sulfate dissimilatory pathway. The canonical dissimilatory sulfate reduction pathway uses an adenylsulfate reductase enzyme to convert APS to sulfite. However, the genome of strain Hulk lacked a clear homolog. Instead, we identified a phosphoadenosine phosphosulfate reductase (PUA_PAPS) (Pdsh_01200 and Pdsh_01605), a bifunctional enzyme that contains non-specific APS reductase and PAPS reductase domains. Sulfite is then reduced to sulfide by a membrane-bound sulfite reductase complex (Pdsh_08990–09020). The sulfide gas can either be expelled (dissimilatory pathway) or assimilated into sulfur-containing compounds such as cysteine via a cysteine synthase (Pdsh_03275).

We also identified a gene cluster (Pdsh_01045–01055) encoding the subunits of a thiosulfate reductase, which catalyzes the reduction of thiosulfate to sulfite in a reaction that generates sulfide gas (Fig. 3c). The sulfite can also be reduced to sulfide by the membrane-bound sulfite reductase of the dissimilatory sulfate reduction pathway (Fig. 3c). Consistent with the genome predictions, strain Hulk grew in media with formate as the electron donor, utilizing sulfate, thiosulfate and sulfite as terminal electron acceptors.

Interestingly, all sulfate/thiosulfate reduction genes of strain Hulk had clear homologs in genes of the type strain Su06^T^ annotated as hypothetical proteins, although Su06^T^ reportedly cannot grow with elemental sulfur and thiosulfate [3]. For example, the gene cluster encoding the dissimilatory sulfite reductase of strain Hulk is also present in the type strain Su06^T^ (Pyrde_0485–0492). The first five genes in the Su06^T^ cluster (Pyrde_486–490) are 100% identical to the homologues in strain Hulk (Pdsh_08990–09010), whereas the last two genes (Pyrde_490 and 491) are 99 and 98% identical to Pdsh_09015 and Pdsh_09020 in the genome of strain Hulk. This suggests that the reduction of sulfite, and perhaps of other sulfur-containing compounds, may be an unsuspected respiratory capability of the type strain Su06^T^ as well. If so, the metabolic hallmark of the family 10.1601/nm.55 is, as proposed earlier [1], the ability of these hyperthermophilic archaea to use sulfur-containing electron acceptors.

## Conclusions

10.1601/nm.28674 strain Hulk is the first hyperthermophilic archaeon able to support growth through the reduction of iron, nitrate, and sulfur-containing compounds. Like most other members of the family 10.1601/nm.55 [1, 3], strain Hulk grows autotrophically with H_2_. However, it is the first member of the family reported to use formate and sugars as electron donors. It also shares with two species in the family, 10.1601/nm.58 AV2^T^ [4] and 10.1601/nm.61 PLM1–5^T^ [5], the ability to use peptides as electron donors. The annotated genome revealed the molecular basis for such remarkable metabolic versatility, with numerous pathways for autotrophic and heterotrophic growth using electron acceptors that are abundant in hydrothermal vent environments. This suggests that hyperthermophilic genomes are under selective pressure to maximize the use of the available resources.

## Abbreviations

AQDS: anthraquinone-2,6-disulfonate
DHAP: dihydroxyacetone phosphate
DMSO: dimethyl sulfoxide

## References

[1] Kashefi K. Hyperthermophiles: metabolic diversity and biotechnological applications. In: P. AR, editor. Extremophiles: microbiology and biotechnology. Norfolk: Caister Academic Press; 2012. p. 183–231.

[2] Blöchl E, Rachel R, Burggraf S, Hafenbradl D, Jannasch HW, Stetter KO (1997). *Pyrolobus fumari*, gen. And sp. nov., represents a novel group of archaea, extending the upper temperature limit for life to 113°C. Extremophiles.

[3] Lin TJ, El Sebae G, Jung J-H, Jung D-H, Park C-S, Holden JF. *Pyrodictium delaneyi* sp. nov., a hyperthermophilic autotrophic archaeon that reduces Fe(III) oxide and nitrate. IJSEM. 2016;(66):3372–6. 10.1099/ijsem.0.001201 PMC6092747 27260263

[4] Pley U, Schipka J, Gambacorta A, Jannasch HW, Fricke H, Rachel R (1991). *Pyrodictium abyssi* sp. nov. represents a novel heterotrophic marine archaeal hyperthermophile growing at 110 °C. System Appl Microbiol.

[5] Zillig W, Holz I, Janekovic D, Klenk HP, Imsel E, Trent J (1990). *Hyperthermus butylicus*, a hyperthermophilic sulfur-reducing archaebacterium that ferments peptides. J Bacteriol.

[6] McCollom TM, Seewald JS (2001). A reassessment of the potential for reduction of dissolved CO2 to hydrocarbons during serpentinization of olivine. Geochim Cosmochim Acta.

[7] Konn C, Magnér J, Charlou J, Holm NG, Alsberg T (2015). A method for detection of trace concentrations of underivatized amino acid in hydrothermal fluids by ion-pairing. Am J Analyt Chem.

[8] Tor JM, Amend JP, Lovley DR (2003). Metabolism of organic compounds in anaerobic, hydrothermal sulphate-reducing marine sediments. Environ Microbiol.

[9] Lang SQ, Butterfield DA, Schute M, Kelley DS, Lilley MD (2010). Elevated concentrations of formate, acetate and dissolved organic carbon found at the lost City hydrothermal field. Geochim Cosmochim Acta.

[10] Kashefi K, Tor JM, Holmes DE, Gaw van Praagh C, Reysenbach A-L, Lovley DR. *Geoglobus ahangari*, gen. Nov., sp. nov., a novel hyperthermophilic archaeon capable of oxidizing organic acids and growing autotrophically on hydrogen with Fe(III) serving as the sole electron acceptor. Intern J System Bacteriol 2002;52:719-728. 10.1099/00207713-52-3-719 12054231

[11] Rodriguez-R LM, Konstantinidis KT. The enveomics collection : a toolbox for specialized analyses of microbial genomes and metagenomes. Peer J Preprints. 2016;

[12] Zhang W, Du PC, Zheng H, Yu WW, Wan L, Chen C (2014). Whole-genome sequence comparison as a method for improving bacterial species definition 75-78. J Gen Appl Microbiol.

[13] Manzella MP, Reguera G, Kashefi K (2013). Extracellular electron transfer to Fe(III) oxides by the hyperthermophilic archaeon *Geoglobus ahangari* via a direct contact mechanism. Appl Environ Microbiol.

[14] Gavrilov SN, Lloyd JR, Kostrikina NA (2012). I. SA. Fe(III) oxide reduction by a gram-positive thermophile: physiological mechanisms for dissimilatory reduction of poorly crystalline Fe(III) oxide by a thermophilic gram-positive bacterium *Carboxydothermus ferrireducens*. Geomicrobiol J.

[15] Bennett S (2004). Solexa Ltd. Pharmacogenomics.

[16] Aronesty E. Command-line tools for processing biological sequencing data. ea-utils. Durham: Expression Analysis; 2011. http://code.google.com/p/ea-utils.

[17] Zerbino DR, Birney E (2008). Velvet: algorithms for de novo short read assembly using de Bruijn graphs. Genome Res.

[18] Soueidan H, Maurier F, Groppi A, Sirand-Pugnet P, Tardy F, Citti C (2013). Finishing bacterial genome assemblies with mix. BMC Bioinformatics.

[19] Aziz RK, Bartels D, Best AA, DeJongh M, T. D, Edwards RA, et al. The RAST Server: rapid annotations using subsystems technology. BMC Genomics 2008;9:75. 10.1186/1471-2164-9-75 PMC2265698 18261238

[20] Delcher AL, Bratke KA, Powers EC, Salzberg SL (2007). Identifying bacterial genes and endosymbiont DNA with Glimmer. Bioinformatics.

[21] Markowitz VM, Chen IM, Palaniappan K, Chu K, Szeto E, Pillay M (2014). IMG4 version of the integrated microbial genomes comparative analysis system. Nucleic Acids Res.

[22] Bagos PG, Nikolaou EP, Liakopoulos TD, Tsirigos KD (2010). Combined prediction of tat and sec signal peptides with hidden Markov models. Bioinformatics.

[23] Krogh A, Larsson B, Von Heijne G, Sonnhammer EL (2001). Predicting transmembrane protein topology with a hidden Markov model: application to complete genomes. J Mol Biol.

[24] Angiuoli SV, Gussman A, Klimke W, Cochrane G, Field D, Garrity GM (2008). Toward an online repository of standard operating procedures (SOPs) for (meta) genomic annotation. OMICS J Integrative Biol.

[25] Brügger K, Chen L, Stark M, Zibat A, Redder P, Ruepp A (2007). The genome of *Hyperthermus butylicus*: a sulfur-reducing, peptide fermenting, neutrophilic Crenarchaeote growing up to 108 °C. Archaea.

[26] Yamazaki S, Yamazaki J, Nishijima K, Otsuka R, Mise M, Ishikawa H (2006). Proteome analysis of an aerobic hyperthermophilic crenarchaeon, *Aeropyrum pernix* K1. Mol Cell Proteomics.

[27] Kelman LM, Kelman Z (2014). Archaeal DNA replication. Annu Rev Genet.

[28] Gao F, Luo H, Zhang C-T (2013). DoriC 5.0: an updated database of oriC regions in both bacterial and archaeal genomes. Nucleic Acids Res.

[29] Bräsen C, Esser D, Rauch B, Siebers B (2014). Carbohydrate metabolism in Archaea: current insights into unusual enzymes and pathways and their regulation. Microbiol Mol Biol Rev.

[30] Hu Y, Holden JF (2006). Citric acid cycle in the hyperthermophilic archaeon *Pyrobaculum islandicum* grown autotrophically, heterotrophically, and mixotrophically with acetate. J Bacteriol.

[31] Amend JP, Amend AC, Valenza M (1998). Determination of volatile fatty acids in the hot springs of Vulcano, Aeolian Islands, Italy. Org Geochem.

[32] Zeng Y, Liu J (2000). Short-chain carboxylates in fluid inclusions in minerals. Appl Geochem.

[33] Campbell BJ, Cary SC (2004). Abundance of reverse tricarboxylic acid cycle genes in free-living microorganisms at deep-sea hydrothermal vents. Appl Environ Microbiol.

[34] Orita I, Sato T, Yurimoto H, Kato N, Atomi H, Imanaka T (2006). The ribulose monophosphate pathway substitutes for the missing pentose phosphate pathway in the archaeon *Thermococcus kodakaraensis*. J Bacteriol.

[35] Welhan JA, Craig H (1979). Methane and hydrogen in East Pacific rise hydrothermal fluids. Geophys Res Lett.

[36] Mardanov AV, Slododkina GB, Slobodkin AI, Beletsky AV, Gavrilov SN, Kublanov IV (2015). The *Geoglobus acetivorans* genome: Fe(III) reductio, acetate utilization, autotrophic growth, and degradation of aromatic compounds in a hyperthermophilic archaeon. Appl Environ Microbiol.

[37] Kashefi K, Holmes DE, Lovley DR, Tor JM, WSD W, EF DL, Kelley DS, Baross JA, Cary SC (2004). Potential importance of dissimilatory Fe(III)-reducing microorganisms in hot sedimentary environments. The subseafloor biosphere at mid-ocean ridges.

[38] Kashefi K, Lovley DR (2003). Extending the upper temperature limit for life. Science.

[39] Weber KA, Achenbach LA, Coates JD (2006). Microorganisms pumping iron: anaerobic microbial iron oxidation and reduction. Nat Rev Microbio.

[40] Friedrich T, Scheide D (2000). The respiratory complex I of bacteria, archaea and eukarya and its module common with membrane-bound multisubunit hydrogenases. FEBS Lett.

[41] Vignais PM, Billoud B, Meyer J (2001). Classification and phylogeny of hydrogenases. FEMS Microbiol Rev.

[42] Forzi L, Sawers RG (2007). Maturation of [NiFe]-hydrogenases in *Escherichia coli*. Biometals.

[43] Böck A, King PW, Blokesch M, Posewitz MC (2006). Maturation of hydrogenases. Adv Microb Physiol.

[44] Chan K-H, Lee K-M, Wong K-B (2012). Interaction between hydrogenase maturation factors HypA and HypB is required for [NiFe]-hydrogenase maturation. PLoS One.

[45] Robson R. Biodiversity of hydrogenases In: Cammack R, Frey M, Robson R, editors. Hydrogen as a fuel : learning from nature. New York Taylor & Francis; 2001. p. 9–32-261.

[46] Yernool DA, McCarthy J, Eveleigh DE, Bok J-D (2000). Cloning and characterization of the glucooligosaccharide catabolic pathway β-glucan glucohydrolase and cellobiose phosphorylase in the marine hyperthermophile *thermotoga neapolitana*. J Bacteriol.

[47] Saha BC, Mathupala SP, Zeikus JG, Himmel ME (1991). Comparison of amylopullulanase to α-amylase and pullulanase. Enzymes in biomass conversion. ACS symposium series.

[48] Lairson LL, Henrissat B, Davies GJ, Withers SG (2008). Glycosyltransferases: structures, functions, and mechanisms. Annu Rev Biochem.

[49] Bertoldo C, Antranikian G (2002). Starch-hydrolyzing enzymes from thermophilic archaea and bacteria. Curr Opin Chem Biol.

[50] Allen JW, Harvat EM, Stevens JM, Ferguson SJ (2006). A variant system I for cytochrome *c* biogenesis in archaea and some bacteria has a novel CcmE and no CcmH. FEBS Lett.

[51] Kletzin A, Heimerl T, Flechsler J, van Niftrik L, Rachel R, Klingl A (2015). Cytochromes *c* in Archaea: distribution, maturation, cell architecture, and the special case of *Ignicoccus hospitalis*. Front Microbiol.

[52] Weber KA, Achenbach LA, Coates JD (2006). Microorganisms pumping iron: anaerobic microbial iron oxidation and reduction. Nat Rev Microbiol.

[53] Childers SE, Ciufo S, Lovley DR (2002). *Geobacter metallireducens* accesses insoluble Fe(III) oxide by chemotaxis. Nature.

[54] Rosenberg E, DeLong EF, Lory S, E. S, Thompson F. The prokaryotes. 4 ed. Rosenberg E DE, Thompson F, Lory S, Stackebrandt E., editor. Berlin Heidelberg: Springer-Verlog; 2013 Feb 2013.

[55] Jarrell KF, Bayley DP, Florian V, Klein A (1996). Isolation and characterization of insertional mutations in flagellin genes in the archaeon *Methanococcus voltae*. Mol Microbiol.

[56] Thomas NA, Paon CT, Jarrell KF (2001). Insertional inactivation of the *flaH* gene in the archaeon *Methanococcus voltae* results in non-flagellated cells. Mol Gen Genomics.

[57] Thomas NA, Mueller S, Klein A, Jarrell KF (2002). Mutants in *flaI* and *flaJ* of the archaeon mutants in flaI and flaJ of the archaeon *Methanococcus voltae* are deficient in flagellum assembly. Mol Microbiol.

[58] Thomas NA, Bardy SL, Jarrell KF (2001). The archaeal flagellum: a different kind of prokaryotic motility structure. FEMS Microbiol Rev.

[59] Park H, Lo YC, Lin SC, Wang L, Yang JK, Wu H (2007). The death domain superfamily in intracellular signaling of aposptosis and inflammation. Annul Rev Immunol.

[60] Desmond E, Brochier-Armanet C, Gribaldo S. Phylogenomics of the archaeal flagellum: rare horizontal gene transfer in a unique motility structure. BMC Evol Biol. 2007;7:16. 10.1186/1471-2148-7-106 PMC1914349 17605801

[61] Seyfried WE, Seewald JS, Berndt ME, Ding K, Foustoukos DI. Chemistry of hydrothermal vent fluids from the main Endeavour field, northern Juan de Fuca ridge: geochemical controls in the aftermath of June 1999 seismic events. J Geophys Res Solid Earth. 2003;108(B9):EPM 5–1 (−23).

[62] Sarmiento JL, Simeon J, Gnanadesikan A, Gruber N, Key RM, Schlitzer R (2007). Deep ocean biogeochemistry of silicic acid and nitrate. Glob Biogeochem Cycles.

[63] Cabello PP, Roldán MD, Moreno-Vivián C (2004). Nitrate reduction and the nitrogen cycle in archaea. Microbiol.

[64] Kuypers MMM, Sliekers AO, Lavik G, Schmid M, Jørgensen BB, Kuenen JG (2003). Anaerobic ammonium oxidation by anammox bacteria in the Black Sea. Nature.

[65] Nicol GW, Schleper C (2006). Ammonia-oxidising Crenarchaeota: important players in the nitrogen cycle?. Trends Microbiol.

[66] Francis C, Beman JM, Kuypers MMM (2007). New processes and players in the nitrogen cycle: the microbial ecology of anaerobic and archaeal ammonia oxidation. ISME J.

[67] Bonete MJ, Martínez-Espinosa RM, Pire C, Zafrilla B, Richardson DJ. Nitrogen metabolism in haloarchaea. Saline Syst. 2008;4:9. 10.1186/1746-1448-4-9 PMC2483277 18593475

[68] Gralnick JA, Vali H, Lies DP, Newman DK. Extracellular respiration of dimethyl sulfoxide by Shewanella oneidensis strain MR-1. Proc Natl Acad Sci USA.103(12):4669–74. 10.1073/pnas.0505959103 PMC1450229 16537430

[69] Peck HD (1961). Enzymatic basis for assimilatory and dissimilatory sulfate reduction. J Bacteriol.

[70] Leustek T (2002). Sulfate metabolism. In: Somerville CR, Meyerowitz EM, editors. The Arabidopsis book. 1. April 4, 2002 ed.

[71] Shen Y, Buick R (2004). The antiquity of microbial sulfate reduction. Earth-Sci Rev.

[72] Kumar S, Stecher G, Tamura K (2016). MEGA7: molecular evolutionary genetics analysis version 7.0 for bigger datasets. Mol Biol Evol.

[73] Pruesse E, Peplies J, Glöckner FO (2012). SINA: accurate high-throughput multiple sequence alignment of ribosomal RNA genes. Bioinformatics.

[74] Field D, Garrity G, Gray T, Morrison N, Selengut J, Sterk P (2008). The minimum information about a genome sequence (MIGS) specification. Nat Biotechnol.

[75] Reysenbach A-L. Thermoprotei class. nov. . 2nd ed. Garrity GM, Boone DR, Castenholz RW, editors. New York: Springer; 2001.

[76] Garrity GM, Holt JG, Phylum AI (2001). Crenarchaeota phy. Nov. 2nd ed. Garrity GM, Boone DR, Castenholz RW, editors.

[77] Reysenbach A-L. *Thermoprotei* class. nov. 1–2. in association with Bergey’s Manual Trust. Wiley; 2015.

[78] Huber H, Stetter KO.* Desulfurococcales* ord. nov. 1–2. in association with Bergey’s Manual Trust. Wiley; 2015.

[79] Huber H, Stetter KO. *Pyrodictiaceae*. 1–2. in association with Bergey’s Manual Trust. Wiley; 2015.

[80] Stetter KO, Koning H, Stackebrandt E (1983). *Pyrodictium* gen. Nov., a new genus of submarine disc-shaped sulphur reducing araebacteria growing optimally at 105°C. Syst Appl Microbiol.

[81] Woese CR, Kandler O, Wheelis ML (1990). Towards a natural system of organisms: proposal for the domains Archaea, bacteria, and Eucarya. Proc Natl Acad Sci U S A.

